# A Case of Successful Foraminotomy for Severe Bilateral C5 Palsy following Posterior Decompression and Fusion Surgery for Cervical Ossification of Posterior Longitudinal Ligament

**DOI:** 10.1155/2016/1250810

**Published:** 2016-09-08

**Authors:** Yoshifumi Kudo, Tomoaki Toyone, Toshiyuki Shirahata, Tomoyuki Ozawa, Akira Matsuoka, Yoichi Jin, Katsunori Inagaki

**Affiliations:** ^1^Department of Orthopaedic Surgery, Showa University School of Medicine, 1-5-8 Hatanodai, Shinagawa-ku, Tokyo 142-8666, Japan; ^2^Department of Orthopaedic Surgery, Ebara Hospital, 4-5-10 Higashiyukigaya, Ota-ku, Tokyo 145-0065, Japan

## Abstract

We report a very rare (5~7%) case of bilateral C5 palsy after cervical surgery. A 71-year-old male patient with cervical ossification of posterior longitudinal ligament (OPLL) with foraminal stenosis at bilateral C4/5 underwent posterior decompression and fusion surgery. After surgery, muscle weakness in his both deltoid and biceps was detected and gradually deteriorated to complete paralysis. Postoperative MRI showed sufficient decompression of the spinal cord and posterior shifting. Subsequently, an additional bilateral foraminotomy at C4/5 was performed, with a suspicion that bilateral foraminal stenosis at C4/5 may have been the cause of the paresis. After foraminotomy, muscular contraction was seen in both deltoid and biceps. Finally, complete motor recovery was achieved in a year. Although the gold standard procedure for the prevention and treatment of postoperative C5 palsy has not yet been established, an additional foraminotomy may be recommended for severe C5 palsy in cases of foraminal stenosis even after the occurrence of palsy.

## 1. Introduction

C5 palsy is well known as one of the most common complications of cervical spine surgery [1, 2], and its incidence has been reported as 4.6% (0~30%) [3, 4]. Most of the paresis (93~95%) occur unilaterally, but the remaining (5~7%) have developed bilaterally [5, 6]. Bilateral cases are very rare and only few reports have been described in detail before [7, 8]. Although there are many reports describing C5 palsy, its pathomechanisms are still controversial [4, 9–12] and prevention of C5 palsy has not yet been established. We encountered a patient with progressing bilateral severe C5 palsy following posterior decompression and fusion for cervical ossification of posterior longitudinal ligament (OPLL). In this male patient complete strength recovery as measured by manual muscle testing (MMT) was achieved almost a year after an additional foraminotomy of C4/5. In this report, this rare case of severe bilateral C5 palsy with complete recovery in MMT is presented and its assumed pathomechanisms are discussed.

## 2. Case Presentation

**Figure 1:**
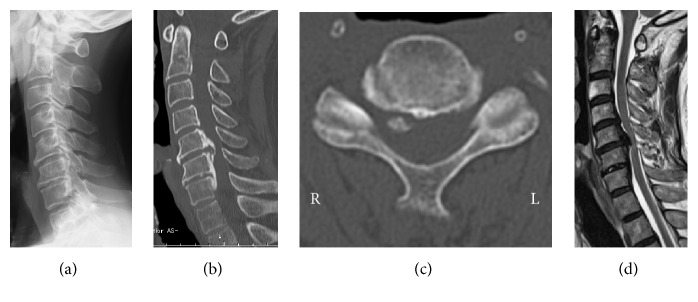
Preoperative radiological findings. (a) and (b): mixed type ossification of the posterior longitudinal ligament was observed on the lateral view of the cervical spine radiograph and sagittal view on computed tomography (CT). Alignment was lordotic and ossification did not exceed the “K-line.” (c): Foraminal stenosis was detected at bilateral C4/5 (1.5 mm on the left side and 2.5 mm on the right side) on axial view CT. (d): high-intensity areas in the spinal cord were not evident on magnetic resonance imaging.

**Figure 2:**
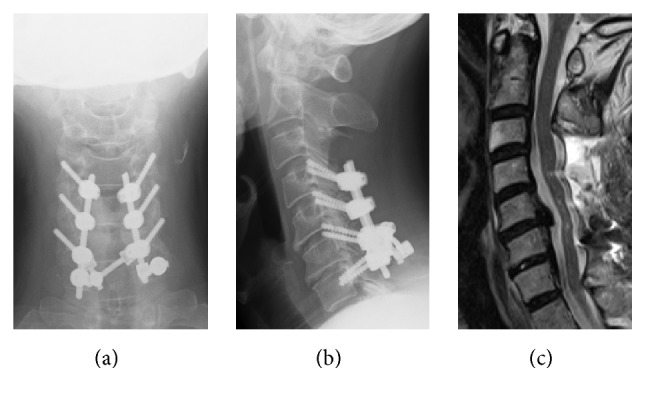
Postoperative X-ray and magnetic resonance imaging. (a) and (b): anteroposterior and lateral view X-ray of the cervical spine. (c): magnetic resonance imaging showed sufficient decompression of the spinal cord and posterior shifting, without high-intensity areas at C3/4.

**Figure 3:**
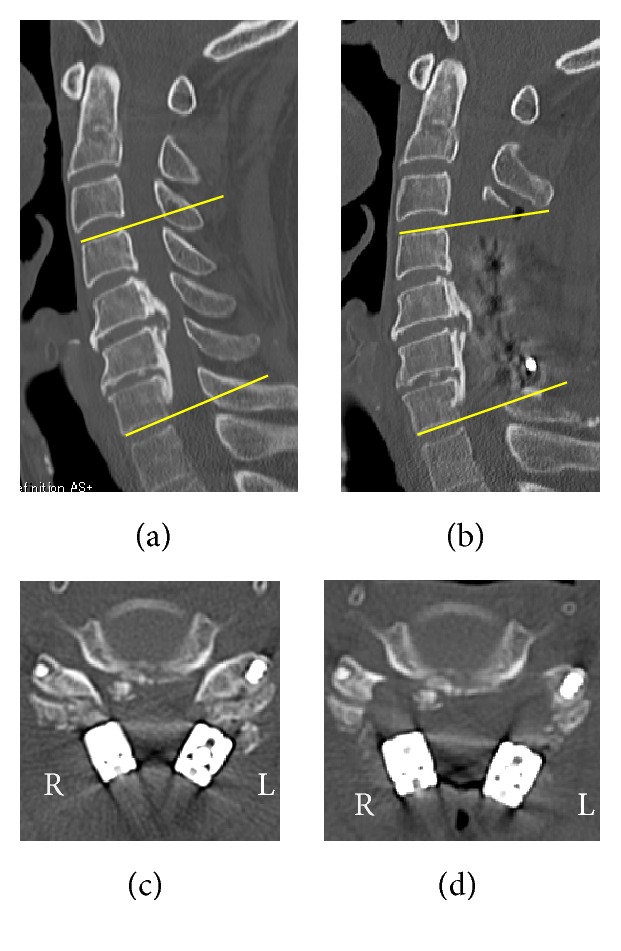
Pre- (a) and postoperative ((b) and (c)) and postadditional foraminotomy (d) computed tomography (CT). (a) and (b): lordosis angle at the operated segment (C4–7) increased by 5 degrees compared to the preoperative angle. (c): there were no changes in anteroposterior diameters of the bilateral C4/5 foramen and no malposition of the screws on postoperative CT. (d): Additional bilateral foraminotomy at C4/5 was performed.

**Figure 4:**
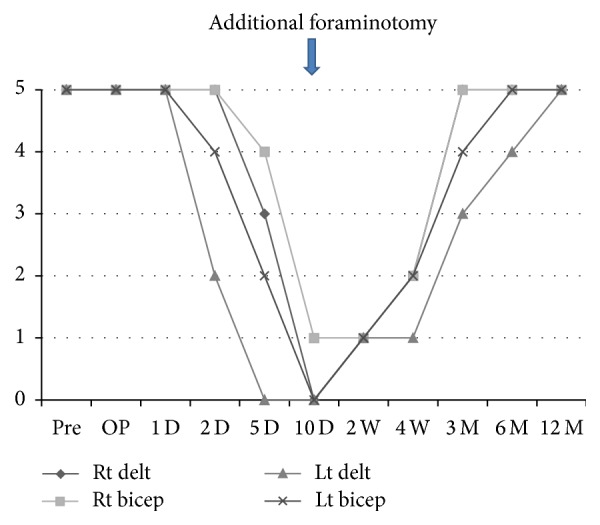
A timeline based graph for the changes of muscle strength (MMT) in bilateral deltoid and biceps. The palsy started on the left side in which foraminal stenosis is more severe on the second day after laminectomy and fusion and then extended on to the right side on the fifth day. Paresis gradually deteriorated and manual muscle testing finally became of grade 0-1 at 10 days after surgery, on which day we performed additional foraminotomy. Muscular contraction was seen at 2 weeks after foraminotomy and gradually improved. The weakness of left deltoid remained. Finally, complete recovery was achieved in a year after additional foraminotomy.

A 71-year-old man complained of unstable gait and numbness in his left upper extremity. On physical examination, numbness was detected in his left upper extremity including C5/6 areas. No muscle weakness was detected including bilateral deltoid and biceps, and deep tendon reflexes were accentuated. Mixed type-OPLL was seen at C4/5/6/7 on the lateral view of the cervical spine X-ray (Figure 1(a)) and sagittal CT (Figure 1(b)). Alignment of the cervical spine was lordotic and ossification did not exceed the “K-Line” [13]. Foraminal stenosis was seen at bilateral C4/5 (1.5 mm on the left side and 2.5 mm on the right side) on CT and high-intensity areas in the spinal cord were not evident on MRI (Figure 1(c)). Posterior decompression (laminectomy) at C3–7 and in situ fusion at C4–7 were performed using instrumentation (Figure 2(a)). On the next day of surgery, this patient started to walk and his unstable gait got better with no muscle weakness on deltoid and biceps. But, on the second day, he started to complain of severe pain in his left scapula, and muscle weakness was detected in his left deltoid and biceps. Postoperative MRI showed sufficient decompression of the spinal cord and posterior shifting (3.8 mm), without high-intensity areas at C3/4 (Figure 2(b)). There were no changes in anteroposterior diameters of the bilateral C4/5 foramen and no malposition of the screws on postoperative CT. Although we performed posterior fusion in situ, lordotic angle at the operated segment (C4–7) increased by 5 degrees compared to the preoperative angle (Figures 3(a), 3(b), and 3(c)). Five days after surgery, he recognized severe pain in his right scapula and muscle weakness in his right deltoid and biceps. Paresis in bilateral deltoids and biceps gradually deteriorated and MMT finally became of grade 0~1 ten days after surgery. Subsequently, we decided to perform an additional bilateral foraminotomy at C4/5 (Figure 3(d)), with a suspicion that foraminal stenosis may have been the cause of the paresis. However, no remarkable change was seen immediately after foraminotomy. One week after the additional operation, electromyographic (EMG) studies were performed. Acute denervation patterns in bilateral C5 > C6 muscle groups were detected with muscular activities remaining in the deltoid and biceps. The patient underwent physical therapies of muscle strengthening exercise and range of motion exercise of the shoulder and elbow joints. Two weeks after foraminotomy, muscular contraction was seen in both deltoid and biceps, followed by grade 2 recovery in these muscles at four weeks. Muscle strength in bilateral biceps and right deltoid recovered completely 3 month after foraminotomy, but grade 3 muscle weakness remained in the left deltoid. Finally, complete motor recovery was achieved in a year. The changes of muscle strength in bilateral biceps and deltoid were shown in a timeline based graph (Figure 4).

## 3. Discussion

Postoperative C5 palsy is a well-recognized complication of cervical decompression surgery. The incidence of C5 palsy is around 4.6% (0~30%) in patients who receive laminoplasty, and bilateral cases are very rare (5%). Although many authors have suggested the mechanisms of C5 palsy, major mechanisms are as follows: (1) nerve root traction as “tethering phenomenon” [2, 4, 9, 14, 15] and (2) disorders occurring at the spinal cord [10–12], but controversies still remain. “Tethering phenomenon” is the hypothesis that tethering of the nerve root might cause C5 palsy as a result of posterior shift of the spinal cord in association with anchoring of the nerve root at the edge of the superior facet. Some authors have proposed that OPLL and foraminal stenosis at C4/5 could be the risk factors of C5 palsy in radiographic analysis [6, 16–18] and prophylactic foraminotomy could prevent postoperative C5 palsy [4, 12, 17, 19].

**Figure 5:**
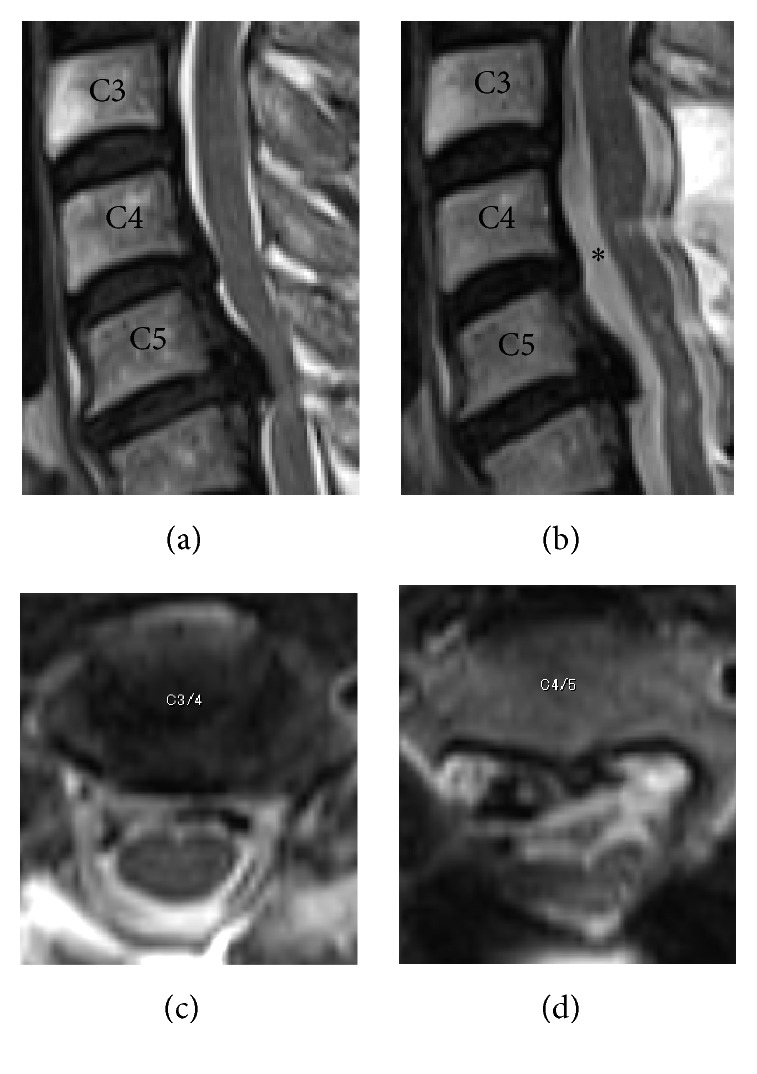
Pre- and postoperative magnetic resonance imaging. (a) and (b): posterior shift of the spinal cord at C4/5 (*∗*) was 3.8 mm. (c) and (d): sufficient decompression of the spinal cord was achieved and high-intensity areas in the spinal cord at C3/4 were not detected.

Matsunaga et al. [17] have reported that there was a significant difference in anteroposterior diameters of the C4/5 foramen between the palsy side (2.3 mm) and the side without palsy (3.3 mm) and have recommended foraminotomy for cases when the diameter is less than 2.5 mm. Nakashima et al. [20] have described that the cut-off values of the pre- and postoperative widths of the C5 intervertebral foramen for C5 palsy were 2.2 and 2.3 mm, respectively. Furthermore, iatrogenic foraminal stenosis could be a cause of C5 palsy after rearrangement of cervical alignment with instrumentation. Takemitsu et al. [21] reported that the risk of developing C5 palsy with instrumentation was 11.6-fold greater than that without instrumentation. In this case, foraminal stenosis was seen at bilateral C4/5 (1.5 mm on the left side and 2.5 mm on the right side) on preoperative CT, while iatrogenic foraminal stenosis was not detected on postoperative CT. Imagama et al. [6] reported that the mean postoperative posterior shift of the spinal cord at C4/5 was 3.9 mm in C5 palsy cases and 3.0 mm in control and have stated that this results in traction and impingement of C5 nerve root as a “tethering phenomenon.” We performed posterior fusion in situ. But, actually, the lordotic angle increased by 5 degrees at C4–7 compared from that of preoperation. This could be a cause of greater posterior shift of the spinal cord (3.8 mm), resulting in the C5 palsy in this case (Figures 5(a) and 5(b)). Some authors have suggested the disorder of the spinal cord, detected by the high frequency of high-intensity area at C3/4 on postoperative MRI, to be the mechanism of C5 palsy [10, 11]. Chiba et al. proposed an etiology that postoperative upper extremity paresis might be associated with deterioration of gray matter such as focal reperfusion injury after acute decompression procedure against ischemic condition of the spinal cord [10]. In our case, no high-intensity area was observed at C3/4 on postoperative MRI, and it is hard to explain that the cause of the C5 palsy originated from the spinal cord even if the paralysis had occurred bilaterally (Figures 5(c) and 5(d)).

We believe that the palsy of this patient might have been caused by multifactorial etiology. Therefore, bilateral foraminal stenosis at C4/5 and posterior shift of the spinal cord which were thought to have been caused by laminectomy and unintentionally gained lordosis might have resulted in the kinking of bilateral C5 nerve roots. Then, the palsy may have started on the left side in which foraminal stenosis is more severe extending on to the right side.

To our knowledge, no report has described severe (grade 0~1) bilateral C5 palsy with full recovery to date. Only two case reports on bilateral grade 2~3/5 C5 palsy have been reported. One is the case report of bilateral C5 palsy following anterior cervical surgery by David and Rao [7], and the other is of anteroposterior decompression and fusion surgery by Jeon and Kim [8]. Spontaneous motor recoveries were achieved with conservative treatment in these cases. In many cases, conservative treatment resulted in complete recovery from postoperative C5 palsy. On the other hand, Imagama et al. reported that 33% of the patients of C5 palsy exhibited residual paralysis, and significantly worse recovery occurred if the patient was severely paralysed at the onset. They also suggested that an additional foraminotomy at an early stage may be useful to treat patients with a severe C5 palsy (MMT = 0 or 1) even after laminoplasty and it may shorten the recovery period [6].

The gold standard procedure for the prevention and treatments of postoperative C5 palsy has not yet been established. Furthermore, the suitable timing of the additional surgery like foraminotomy is debatable. Katsumi et al. presented a prospective study to investigate the effectiveness of prophylactic bilateral C4/5 foraminotomy to prevent postoperative C5 palsy. They reported that prophylactic foraminotomy significantly decreased the incidence of C5 palsy to 1.4% compared with 6.4% in patients without foraminotomy [19]. Practically, prophylactic foraminotomy should be performed when the foraminal stenosis is obvious in preoperative CT. However, if prophylactic foraminotomy prevents postoperative C5 palsy, additional foraminotomy could be effective for recovery even after the occurrence of severe paresis. It might be preferable that surgeons consider the additional foraminotomy in case of progressing severe (MMT0~1) C5 palsy at early stage. Future research is needed to establish the guideline for prevention and treatments for postoperative C5 palsy.

## 4. Conclusion

We experienced a very rare case of severe bilateral C5 palsy following posterior decompression and fusion for cervical OPLL. Multifactorial etiology including foraminal stenosis and posterior shift of the spinal cord due to laminectomy and unintentionally gained lordosis with posterior fusion may be responsible causes for the palsy. An additional foraminotomy at early stage may be recommended for severe C5 palsy in cases of foraminal stenosis even after the occurrence of severe palsy.

## Abbreviations

OPLL: Ossification of the posterior longitudinal ligament
MMT: Manual muscle testing
MRI: Magnetic resonance imaging
CT: Computed tomography.
